# TRASER - Total Reflection Amplification of Spontaneous Emission of Radiation

**DOI:** 10.1371/journal.pone.0035899

**Published:** 2012-04-27

**Authors:** Christopher B. Zachary, Morgan Gustavsson

**Affiliations:** 1 Department of Dermatology, University of California Irvine, Irvine, California, United States of America; 2 Rockport Consulting Services, Inc., Newport Beach, California, United States of America; University of Tennessee, United States of America

## Abstract

**Background and Objective:**

Light and lasers in medical therapy have made dramatic strides since their invention five decades ago. However, the manufacture of lasers can be complex and expensive which often makes treatments limited and costly. Further, no single laser will provide the correct parameters to treat all things. Hence, laser specialists often need multiple devices to practice their specialty. A new concept is described herein that has the potential to replace many lasers and light sources with a single ‘tunable’ device.

**Study Design/Material and Methods:**

This device amplifies spontaneous emission of radiation by capturing and retaining photons through total internal reflection, hence the acronym Total Reflection Amplification of Spontaneous Emission of Radiation, or TRASER.

**Results:**

Specific peaks of light can be produced in a reproducible manner with high peak powers of variable pulse durations, a large spot size, and high repetition rate.

**Conclusion:**

Considering the characteristics and parameters of Traser technology, it is possible that this one device would likely be able to replace the pulsed dye laser and many other light based systems.

## Introduction

This paper will introduce a new concept in light amplification and delivery for medical, domestic, and industrial uses. It will also define peak wavelengths, pulse-widths, pulse modes and fluence levels of spontaneous emission from multiple dyes. Trasers can be defined according to their excited media, and are known as pulsed dye Trasers or crystal Trasers, the latter for the solid crystal dye media grown specifically for this device.

For decades, laser and light sources have provided tremendously important benefits to industry, medicine and mankind. Since Anderson and Parrish developed the concept of selective photothermolysis, laser surgeons have been able to match the wavelength and pulse-width to an anatomical structure, and to selectively heat this without collateral damage [1]. The ability to amplify light by stimulated emission of radiation is the cornerstone of laser technology; it is this amplification (along with collimation, monochromicity, and in-phase characteristics) that differentiates it from other light sources.

**Figure 1:**
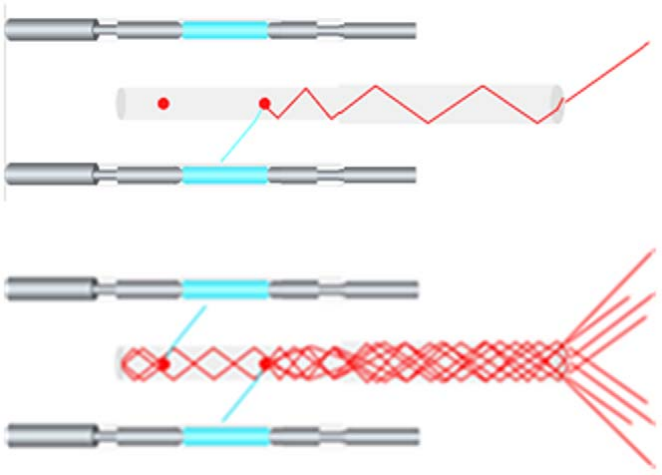
The Traser contains an internally reflecting body hosting a fluorescent substance, “dye cell” (center); flash lamps (above and below); a rear mirror (end left); an optional output waveguide (right); an optional reflector cavity housing the light source and the internally reflecting body (not shown). Photons from the flashlamps excite the fluorescent material within the dye cells, which spontaneously emit a different narrow spectrum of light. Typically, the dye cell traps 45–62% of the spontaneous emission by internal reflection. The internally reflected photons propagate axially along the length of the dye cell or crystal in both directions. At the proximal end of th e cell is placed a mirror, redirecting the light forward. The light is passively coupled out at the distal end of the cell. A wave guide is used to target the treatment area.

The development of the Traser represents a novel and efficient method to induce, capture and amplify light, and to do so with a tunable range of wavelengths. A Traser is not a laser; it is fundamentally different, and, in many ways, much simpler in design. A Traser utilizes the energy from, for example, a flashlamp to induce the spontaneous emission of photons from a fluorescent dye in solution, or ions impregnated in a crystal structure. The narrow band of fluorescence has a peak of intensity that depends on the Stokes shift characteristics of selected fluorescent media within the arc lamp configuration [2] The light generated can be tuned from UVA to near infrared (NIR). As opposed to a laser, a Traser has no optical resonator, no output coupler, and no stimulated emission in the classical sense. Unlike a laser, a Traser induces light that is non-coherent and non-collimated. And in contrast to the intense pulsed light (IPL), none of the emitted photons are derived from the flashlamp, there are no filters, and no filter technology (Figs. 1a, 1b). Amplification of light is achieved by capturing and trapping 46–61% of spontaneously induced photons within a liquid jet or solid body of a high refractive index and the option for special coating so as enhance total internal reflection (TIR).

Traser technology is simple in design and generally more ecofriendly than some of the devices that it could possibly replace. In this regard, Traser technology does not preclude the use of flammable and toxic solvents or toxic additives, but to date Trasers have been operated with water and no additives at all. Many of the fluorescent dyes chosen thus far are non-toxic and, in any event, the user never comes into contact with these dyes. All currently selected dyes are water soluble, and do not require hazardous solvents or additives. When required, they are reclaimed by a filter loop, which in less than a minute can completely eliminate the dye and purify the circulating water. This water is not only reconstituted with new dyes within the Traser circulation cavity, but is also used to cool the device. Consumables would likely be minimal with the life of the flashlamp and dye being years, depending on utilization. Hence, the cost of operation of a Traser should be modest, both for medical facilities and for patients. The likely versatility of this technology – being able to produce UVA, blue, green, orange, red, and NIR – will mean that one Traser inducing an array of tunable wavelengths might replace a number of separate devices currently required for clinical practice.

## Materials and Methods

Three highly fluorescent Traser dyes (pyrromethene 556, rhodamine 590, and sulforhodamine 640 chloride (Exciton, Inc. Dayton, Ohio)) were tested in a standardized arrangement.

For optimum power, each of these dyes was dissolved, one at the time, at a specific concentration in distilled water and recirculated through a cylindrical dye cell comprising 5/7×84 mm N-LASF43 glass (Schott, GmbH, Mainz, Germany). The dye cell was irradiated with light from two xenon flashlamps (Applied Photon Technology, Inc. Hayward, CA) of 5/6.2×80 mm and with a fill pressure of 1400 Torr. The lamps were dressed with cooling water flow jackets of cerium doped glass (Haereus Quarzglas GmbH & Co. KG, Kleinostheim, Germany). The lamp and flow-jacket assemblies and the dye cell were enclosed in a race track shaped close-coupling reflector of unprotected silver sheet (SurePure, Inc. Florham Park, NJ), the latter providing a modest 80% specular reflectivity at 534 nm. A silver mirror (ThorLabs, Inc. Newton, NJ, P/N PF03-03-P01) was located 0.5 mm from the proximal end of the dye-cell, the gap being occupied by the dye solution flow chamber. The light was passively coupled into a non-oriented sapphire crystal waveguide, with a diameter of 8 mm (0.5 cm^2^ cross section) and length of 85 mm. The barrel of the sapphire waveguide was industrial polished and coated with Polytetrafluoroethylene (PTFE). The fluorescent dye solvent was cooled to 1–3°C using a 700 W inert Peltier cooler (Rockport Consulting Services, Inc. Newport Beach, CA). An inert 2 µm particle filter (McMASTER-CARR, Inc. Princeton, NJ) was utilized in the dye circulation loop, and circulated at a speed of 2.5 L/min. The cooled dye solution provided initial refrigeration for the sapphire waveguide before excitation, with the contact sapphire treatment tip being at about 1–3°C.

The electronic capacitor bank of 36,600 µF was set at 640 VDC, and each lamp was discharged by separate insulated gate bipolar transistor (IGBT) switches. The lamps were simmered at 40 V and 600 mAmp. The lamps were pulsed simultaneously as single pulses up to 2 ms, or in a train formation for longer timeframes with individual pulses of 1 ms duration at a duty cycle of 50% for up to 80 ms. In a few cases, trains comprised pulses of up to 1.5 ms (Table 1). Lamp envelope deterioration was noted at one occasion with pulse train durations longer than 80 ms in response to high wall loading. Rather than complicating the comparison of data by decreasing the capacitor bank voltage or altering the duty cycle, no pulses exceeding 80 ms were tested in this study.

**Table 1:** Comparison of the average fluences in parallel mode of 3 sets of optimized dyes.

Spot Size; 8 mm diameter				PM 556	Rh 590	S.Rh 640 Cl.
				0.15 g/L	0.0375 g/L	0.075 g/L
Parallel mode pulse train						
Pulsewidth	# pulsesper lamp	Sub-incrementPulsewidth	Sub-incrementOff time	Peak @544 nmAverageFluence	Peak @592 nmAverageFluence	Peak @658 nmAverageFluence
ms		Ms	ms	J/cm2	J/cm2	J/cm2
0.5	1	0.5	–	6.9	5.5	4.04
1	1	1	–	12.2	9.7	7.39
2	1	2	–	18.3	16.0	12.39
3	2	1	1	16.5	15.0	11.66
4	2	1.5	1	26.9	21.2	19.08
9	5	1	1	45.2	41.1	32.06
19	10	1	1	81.5	78.8	59.56
39	20	1	1	132.5	128.2	99.81
59	30	1	1	165.8	159.8	127.68
79	40	1	1	187.1	179.8	139.52

PM 556 = pyrromethene 556; Rh 590 = rhodamine 590; S.Rh 640 = sulforhodamine 640 chloride.

The output was measured using a spectrum analyzer (Ocean Optics, Inc., HR4000CG-UV-NIR, Dunedin, Fl), a Pyro-Stack energy meter (Ophir, Ltd., Har Hotzvim, Israel, model L40150ADB-NS, P/N174241A with readout P/N 1Z02512). A photodiode (CVI Melles Griot Laser Group, P/N 13DAS005, Carlsbad, CA) was used to verify the pulsewidth. The laser dyes where evaluated with respect to liquid concentration, light spectrum and light fluence.

## Results

**Figure 2:**
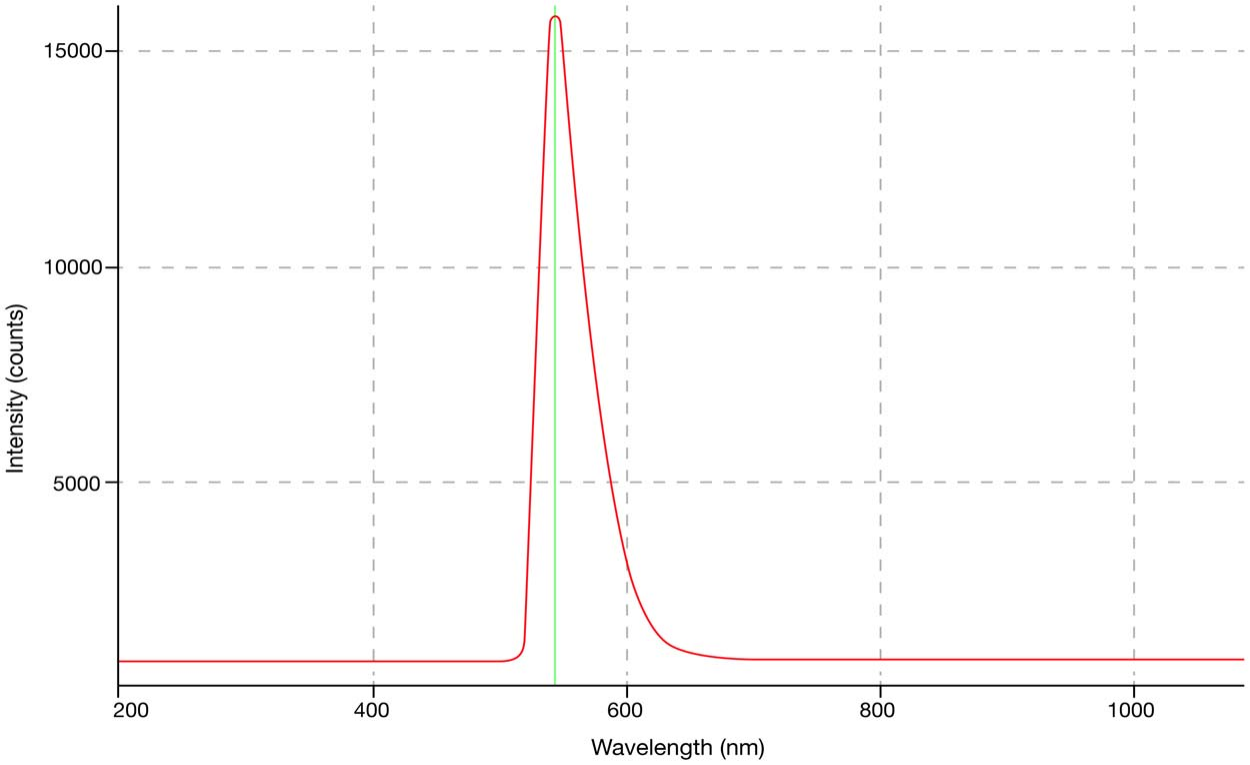
Pyrromethene 556, 0.15 g/L. Peak at 544 nm.

Concentrations from 9.38×10^−4^ g/L to 3000×10^−4^ g/L were evaluated and will be reported separately. Optimal concentrations were established for each dye in relation to power and dye-cell aspect ratios. These ranged from 0.0375 g/L for rhodamine 590 to 0.15 g/L for pyrromethene 556. Wavelength peaks ranged from 533 nm for pyrromethene 556 @ 0.0375 g/L to 662 nm for sulforhodamine 640 chloride @ 0.15 g/L. The optimum power yielding dye concentrations for the three dyes, subject to the dye cell aspect ratio, are found in Table 1 and Figures 2–4. No difference in emission peaks were found with increasing fluence or increasing pulsewidth.

**Figure 3 pone-0035899-g003:**
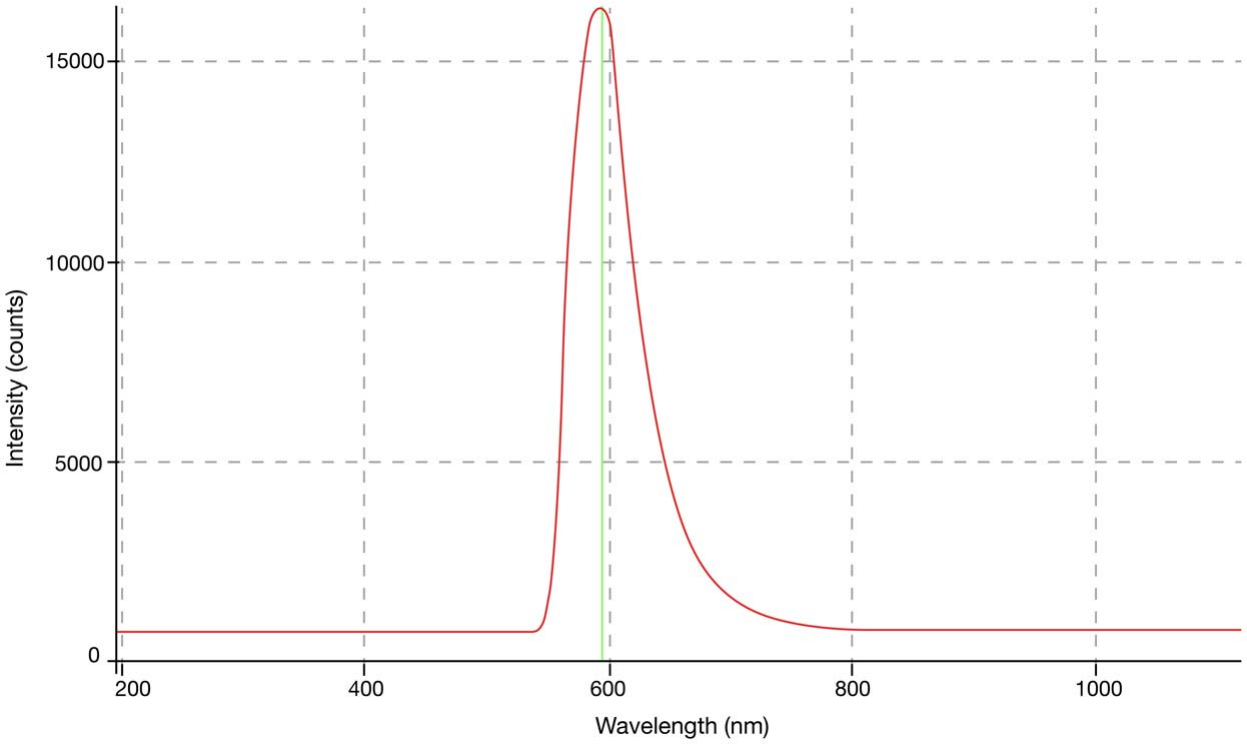
Rhodamine 590, 0.0375 g/L. Peak 592 nm.

**Figure 4:**
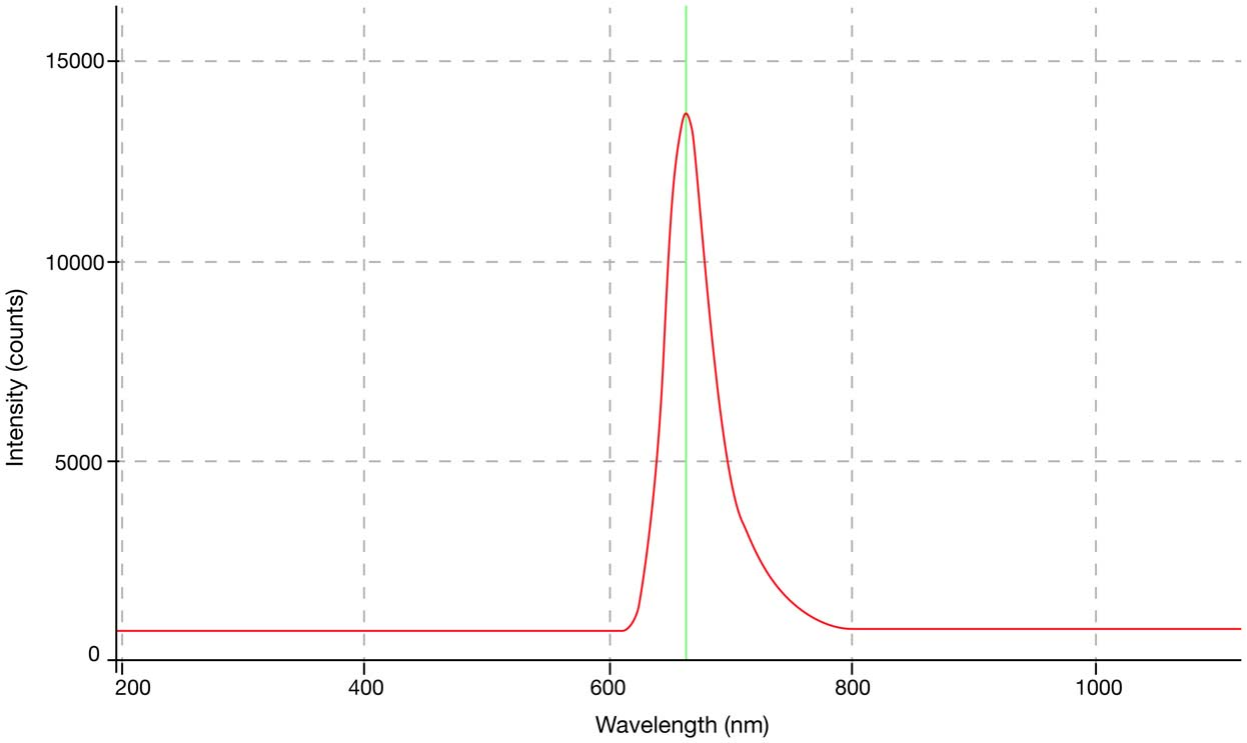
Sulphorhodamine 640, 0.75 g/L. Peak at 658 nm.

Fluences were assessed for all three dyes, and tested in single pulse mode from 0.5–2.0 ms, and in pulse-train mode from 3–79 ms. It should be noted that fluences were achieved well above those normally recommended for clinical use. In the clinical situation, this would be easily moderated by lowering the voltage over the capacitor bank, reducing the duty cycle, or increasing the treatment spot size, with one or all three resulting in lower fluences. For the purposes of this study, diminishing these high fluences by any of the said three options would have made a direct comparison of the dyes impossible at many pulse widths. Table 1 lists the dyes in parallel mode for each dye, and with increasing fluences. For all three dyes and with all pulse trains, the lamp duty cycle is expressed as ‘on’ and ‘off’ times during the pulse-train. The most common lamp duty cycle was 50%, 1 msec on and 1 msec off.

Figs. 2–4 show Traser emission spectra from dyes exhibiting lower to higher fluences, in the same order as in Table 1, including their optimal dye concentrations, given the currently configured dye cell aspect ratio.

**Figure 5:**
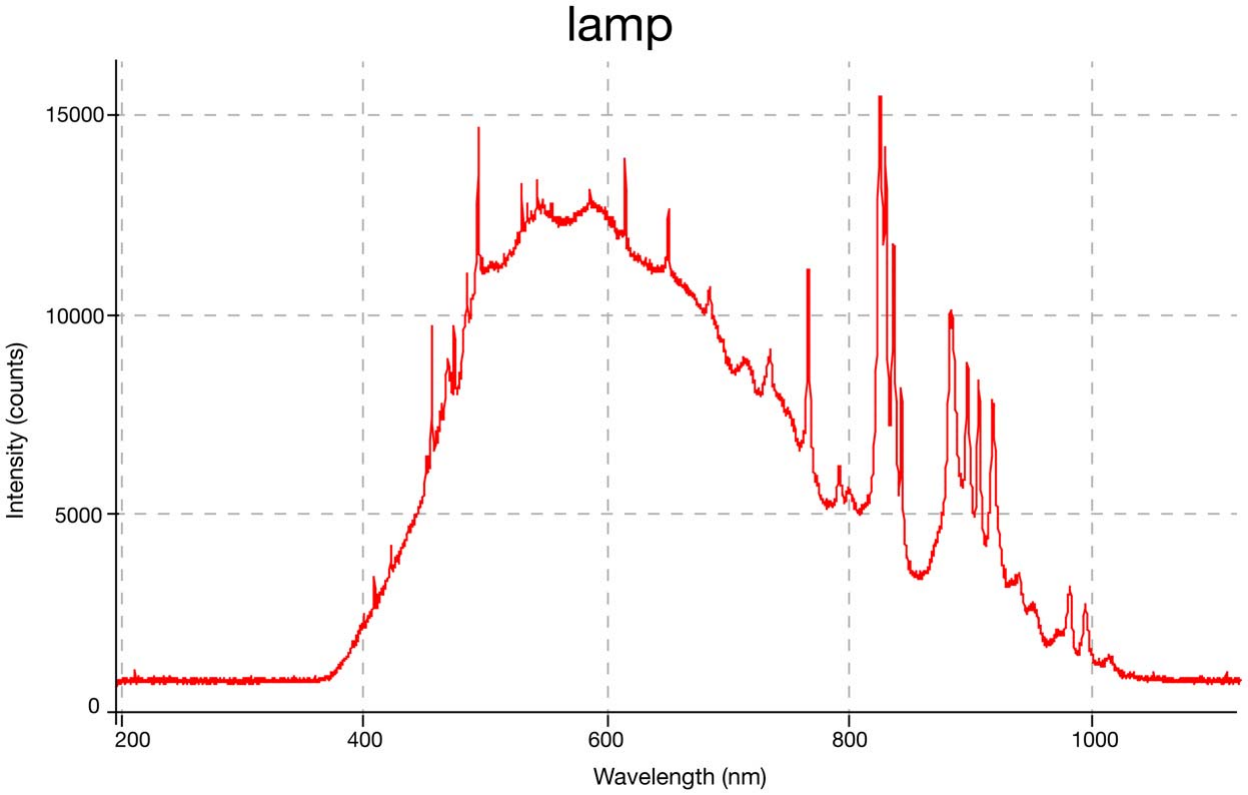
Flashlamp spectrum alone, without dye cell emission.

It was important for us to demonstrate that the typical flashlamp emission spectrum (Fig. 5) did not significantly contaminate the spontaneous emission from the dye cell. On occasions, the flashlamps’ discharge could be identified in the dye emission graphs between 800 and 950 nm, some of which was contamination from minor lamp light leakage into the laboratory, and some of which was refracted at the interface between the water-dye solution and the sapphire rod (Figs. 4,5). Pyrromethene 556 at 0.15 g/L had the highest fluence of the candidate dyes, at which peaks all flashlamp leakage was completely suppressed.

**Figure 6:**
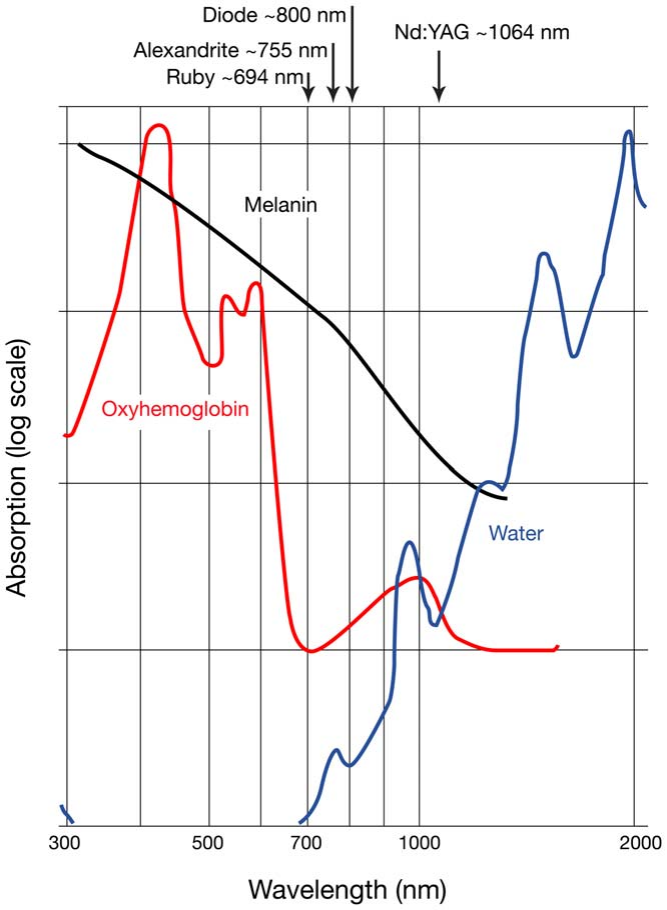
Light absorption by tissue chromophores [1].

As an example of the therapeutic potential for these dyes, the pyrromethene 556 emission spectrum could be nicely superimposed over the absorption spectrum of oxygenated blood (Fig. 6). In Figs. 4 and 6 [3], the sulforhodamine 640 chloride peak is seen to be strategically positioned at 662 nm at the area of greatest discrepancy of absorption between hemoglobin and melanin, clearly indicating hair as a candidate target.

There was no degradation of dyes over the duration of the study. However, over an extended period one could expect degradation, and these data will be presented in a separate paper.

## Discussion

The heart of a Traser is by design simpler than a laser, has fewer components such as an optical resonator or output coupler, and no filters or frequency doubling crystals. It needs no mirror alignment and has no lasing thresholds or population inversions to consider. The output fluence is perfectly linear with the current going through the lamp, and hence the power can be set by a CPU calibration table for the voltage over the capacitor bank. By and large, simpler engineering designs are associated with increased reliability. The few consumables are inexpensive, generally non-toxic, and extremely durable. The amplification process is not one of stimulated emission of radiation. Rather it is based on the trapping of 46–61% of the photons over a predetermined length of a highly reflecting dye cell or solid host. In contrast, the surface of a laser rod is typically non-reflecting in order to prevent parasitic photon angles. Indeed, photons in a laser that propagate at angles other than substantially parallel to the axis of the dye cell or crystal will induce stimulated emission from excited matter in directions that will not benefit the multiplicative build up of collimated photons. In practice, they will decrease the amount of excited molecules or ions available for lasing. As such these photons are called ‘parasitic’, and their angles ‘parasitic angles’. In a crystal laser it is common practice to not polish the barrel of the rod in order to prevent total internal reflection, which would only increase the amount of parasitic photons at parasitic angles.

Spontaneously emitted photons are arrayed in a non-collimated spherical design. Of these, 46–61% are unable to escape the dye cell because of TIR at the surface of the dye-cell or rod. In addition, the dye cell or crystal rod can be designed with appropriate low pass coatings to further improve refraction, and almost completely prevent loss of spontaneous emission.

The specific peak of wavelength output can be varied by altering the concentration of a particular dye, or by utilizing a different fluorescent dye. To remove the existing dye, the solution is passed through a deionizing filter, after which the purified solvent, generally water, is reconstituted with a new dye pellet. This process makes dye changes efficient and integrative, since the circulating dye is also used as a cooling medium for the waveguide. The key to understanding how to optimize the wavelength output is by recognizing that, in response to a flashlamp pulse, fluorescent dyes have a characteristic absorption pattern, and an equally characteristic spontaneous output fluorescence (Stokes shift), which peaks at a specific wavelength, based in part on red shift with increasing dye concentrations.

Design of the dye cell determines the total energy output from the device. A 5 mm diameter dye cell may generate the same fluence as a 12 mm diameter dye cell, but the total energy produced with the 12 mm diameter cell will be significantly higher. This will have implications for device engineers, as there is a maximal optimal diameter of the dye cell after which the fluence will start to decrease with any further increase in diameter. This is expressed as the relationship between the diameter and the length of the dye cell, the so-called ‘aspect ratio’. In general, a larger diameter dye cell will allow a larger treatment spot size, while maintaining fluence. Decisions with regard to intended use outcomes will drive the dimensions of the dye cell and the dye concentration.

There is some confusion about the concept of ‘amplification’. The laws of nature require that, in both cases, laser and Traser, there is no true ‘amplification’ of light energy. On the contrary, there is a weakening of photonic energy during these processes, wherein light is merely ‘concentrated’ over a predetermined area. A Traser amplifies light no more and no less than a laser. In both cases, for every one photon ‘out’ (with the energy E), there has been one photon ‘in’ (with energy > E) from the so-called pump source, for instance a flash-lamp. This is also the reason why photons *exiting* a Traser or a laser have longer wavelength than those *entering* the fluorescent media in these devices.

In the case of a crystal laser or a dye-laser there is.

1. a first photon, derived from a flash-lamp, (Lamp Derived Photon 1 - LDP1) excites a fluorescent molecule (FM1), that spontaneously emits a fluorescence derived photon (FDP1) and
2. a second flash-lamp derived photon (LDP2) excites a different fluorescent molecule (FM2) which will produce its own photon FDP2. However, while FM2 is still in its excited state, an FDP1 hits it and as a result two photons, FDP1’s identical successor, namely FDP3, and FDP2 are launched at the same moment. That is what is referred to as stimulated emission. The fact that both photons are unidirectional (collimated) is the critical mechanism by which laser light is concentrated over a predetermined area. While this has significant benefits in terms of delivery mechanisms, collimation is not amplification. It is simply confinement of photons over a smaller area than otherwise would have been the case, that is, ‘concentration of light’ rather than ‘amplification of light’.

Population inversion is a feature of distinction between a laser and a Traser. Mirrors at both ends of a laser cavity augment coherence of the unidirectional FDP1&3 photons in bi-axial directions. With FDP3 and FDP2 photons oscillating between said mirrors, more and more FDP3 and FDP2 are emitted in the same bidirectional axis.

Based on the one photon ‘in’, and one photon ‘out’ concept, what is achieved is a concentration of photons with a certain directional properties. While this might be an oversimplification, lasers do not amplify photonic energy or ever create more photonic energy ‘out’ than was ever pumped ‘in’. On the contrary there is a net loss in energy, photon for photon, of every photon incident to and absorbed by the fluorescent and lasing medium to every photon emitted by a laser as collimated light. In addition to a decrease in photonic energy, there is a loss of fluorescent light emitted in every other but axial directions from the fluorescent medium, and of all the light from the so called pump-source not absorbed and re-emitted by the lasing medium. The electro-optical efficacy of a laser is typically about or less than 1–1.2%.

In the case of a crystal Traser or a dye Traser, the same process described above induces both FDP1 and FDP2 photons, which are confined in and travel through the same predetermined longitudinal volume as do FDP1, FDP2 and FDP3 photons in a flash-lamp “pumped” dye or crystal laser. However they do so at a plurality of angles and are not coherent as in a laser. The mechanism for concentrating light over a predetermined area in a Traser is not based on stimulated emission, but instead on the delta in refractive index of the fluorescent medium’s host and the cladding of it, that is, air. Typically, 46–61%, of all FDP1 and FDP2 photons are salvaged within the dye cell and concentrated over a predetermined area. Note that FDP1 need not transcend into FDP3 for this concentration of energy. In reality, it is almost exclusively FDP1 and FDP2 that are being concentrated over this area.

Although not entirely accurate, when we refer to spatial concentration of photons as ‘amplification’, it serves the purpose of differentiating both the Traser and the laser from other ways of achieving spatial concentration of light, for instance with the use of a focusing lens.

To summarize, the term ‘amplification’ of light has become standard usage among laser engineers and would probably be better understood as ‘concentration’ of light confined over a small area. (The word LCSER doesn’t roll off the tongue quite so easily as LASER, so we assume that ‘amplification’ is going to remain the term du jour). If some are concerned about the use of the term ‘amplification’ in a Traser, then they might have the exact same concern about the use of this word with regard a laser. If a laser amplifies light, then a Traser, using the same principles of quantum mechanics, also amplifies light. While we understand the confusion, it’s a case of semantics.

Given the design of a Traser, it is generally more ecofriendly and safer than corresponding dye lasers. For eco-friendliness we are looking at four components, namely dye, solvent, additive and material handling. The current dye lasers use flammable and toxic organic solvents with additives such as the cyclooctatetraene (COT). By comparison, the Trasers are operated using water as the host (i.e. the solvent) for the Traser dye, and do not require toxic solvent additives. Furthermore, the closed system in a Traser avoids any dye or solvent contact with the operator. A Traser can be operated with a peak at any of the common yellow dye lasers without the use of any toxic solvents, solvent additives, or toxic dyes. Even with the use of rhodamines, with water as a solvent and without any additives, the Traser is ecofriendlier than the corresponding lasers.

To understand the reasons why additives such as COT are required in a dye laser, and not in a dye Traser, we offer the following explanation. In a dye laser system, over time, an increasing portion of the dye molecules, upon excitation, get ‘stuck’ in a non-fluorescing spinning and vibrational state, the so called ‘triplet state’. While in this triplet state, the dye molecules will not support either spontaneous or stimulated emission. To counteract this problem in a dye laser, the population of triplet state dye molecules is commonly reduced by the addition of agents such as COT. This is a potentially irritating agent to the skin, eyes and lungs, is flammable and “readily forms explosive mixtures with air” [4].

Laser engineers tend not to use cladding around the fluorescent media, which consequently results in leakage of light out of the sides of the crystal rod or dye cell. This loss of photonic energy is compensated, to some degree, with a leaking front mirror, a so-called “output coupler”. The overall electro-optical efficacy of a dye laser is typically up to about 1.2% versus typically 2.5% for the Trasers built to date.

The pulsed mechanism in this device can produce individual pulses from 0.15–100 msec, only limited by the plasma growth within the Xenon gas of the lamp, the size of the capacitor bank, and the Traser software.

The Traser emission spectra match hemoglobin’s absorption wavelengths, indicating that a Traser could indeed be harnessed to treat the same vascular conditions as the PDLs by the same selective photothermolysis mechanisms. For superficial port wine stains, the shorter wavelengths with shorter pulse durations may be optimal, and for those with thicker, more nodular problems, the longer wavelengths with longer pulses might be preferred. Further, one can predict good responses in patients with lentigenes and in hair removal.

The current design of a Traser will allow physicians and laser engineers the ability to produce highly variable parameters of wavelength, pulse duration, fluence and spot size. For instance, the current trend by the leading manufacturers of pulsed dye lasers is to deliver individual pulses comprising 6 or 8 micropulses. If a clinician deems this of use, a Traser device could deliver an appropriate peak of energy with the same micropulsing technology. However, some might consider that these high peak power micropulses tend to induce partial vessel disruption, with or without associated purpura [5]. Unless the vessel is totally ‘cooked’, revascularization from residual endothelium might be responsible for recurrence. Further, locally induced angiogenic growth factors may be responsible for the rapid rate of revascularization. Given all this, the treating physician using a pulsed dye Traser could choose true continuous pulses, tunable in wavelength, and in 0.2 msec steps from 0.2–100 msec.

One example of a prime target for a Traser would be hair removal. One could, for instance, resurrect the concept of using light in the upper 600 s nm region. The early publications concerning hair removal utilized ruby lasers (694 nm), and the results were impressive [6]. That they went out of style was unrelated to their efficacy, but rather their burdensome maintenance, unreliability and temperamental behavior, and a maximal pulse being limited to 3–4 msec. Our studies indicate that high peak powers can be obtained at these wavelengths even over long 10–100 msec pulses, and as such this wavelength may well be considered more favorably in the future for hair removal. Clearly darker skin types would require reduced fluence, efficient cooling and longer pulse duration, but Asians and Caucasians in particular could benefit from the significant melanin/hemoglobin absorption differential with this wavelength [7].

One practical question relates to the application of this device on the skin. The treatment tip would have to be in contact with the skin because of the divergent nature of the wave-front. The proposed hand piece is envisaged as a 1–5 cm (in length) interchangeable tip with contact cooling. The smallest spot would be 1 mm. This might be further reduced by a truncating block or spatial filter located at the proximal end of the distal treatment tip.

Given that a pulsed dye Traser is tunable, is should be able to mimic devices from below the 532 nm (green) to the near infrared wavelength simply by changing the dye kit. The durability of the flashlamps in a Traser are likely to be significant, given the relatively low peak powers and longer individual pulse durations (0.45 msec) and the consequent wear and tend on both the lamps. The same would be true for the dye.

Trasers should compare well with an intense pulse lights (IPL). Trasers have chromophor-absorption selective spectra that are quite similar to those of lasers. Since there is less interference by wavelengths induced by less selective absorption, the fluences required to induce the same peak power output is reduced. Even with short pulses, Trasers are capable of producing high peak powers within a narrow wavelength spectrum, a feature impossible in the case of IPLs. The fluences delivered from a Traser are in parity with, or exceed, those delivered by a comparable laser, even at short pulse-durations. This is even more evident when compared to an IPL.

Lasers are generally considered by the public to be state-of-the-art devices for achieving optimal outcomes in a wide array of matters. However in the medical world, there is a debate about which is optimal, a laser or an intense pulsed light, for conditions such as port wine stains, telangiectases, skin pigmentation and hair removal. In practice both have their places [8].

The Earth is drenched by a wide array of wavelengths of light and our diverse biological nature is engineered to respond to many of them. It’s not dependent upon a few single wavelengths of light. As an example, hemoglobin has a broad range of light absorption, and yet unless we are using a filtered intense pulsed light, we generally offer the hemoglobin just one wavelength for a therapeutic response. One could argue that a pulse dye Traser’s broader range of wavelengths will have an enhanced range of penetration depth and internal scattering, both of which could augment heating of the more superficial and deeper vascular components.

When the pulsed dye laser was first produced, a wavelength of 577 nm was deliberately engineered because it was considered optimal for an absorption peak of oxygenated haemoglobin [9]. However, this wavelength was so well absorbed by hemoglobin that it failed to coagulate the entire diameter of the blood vessel. Oon Tien Tan et al experimented with wavelengths above and below the 577 nm peak, and found that moving up to 585 nm decreased the absorption but doubled the depth of penetration, two essential measures which enhanced the efficacy of the pulsed dye laser (PDL) [10]. In an attempt to further augment the heating of a heterogeneous group of vessels, most PDLs now deliver a train of 595 nm pulses comprised of up to eight 0.45 msec pulses over a period of up to 40 msec [11]. A pulsed dye Traser can be engineered to emulate the PDL with multiple short pulses, or to deliver single true continues pulses of up to 100 msec.

This paper has concentrated on the science behind the concept of a Traser. There is no such device currently available or cleared by the FDA. The significance of a single device with the functionality of many will be intriguing to clinicians and engineers. It could represent an alternative generation of devices to be used in clinical medicine.
